# Living with type 1 diabetes and schooling among young people in Ghana: a truism of health selection, inadequate support, or artefactual explanation of educational inequalities?

**DOI:** 10.1186/s12889-024-18590-y

**Published:** 2024-04-24

**Authors:** Bernard Afriyie Owusu, David Teye Doku

**Affiliations:** Department of Population and Health, University of Cape Coast, Cape Coast, Ghana

**Keywords:** Type 1 diabetes mellitus, Youth, Social support, Health inequalities

## Abstract

**Introduction:**

Type 1 diabetes mellitus (T1DM) is mostly diagnosed among young people. Despite the evidence that T1DM is disruptive, and affects individuals’ health and cognitive ability, there is dearth of knowledge on the impact of T1DM on schooling in LMICs including Ghana. In this research, we explored the impact of T1DM on the schooling of young people living with the disease, and discussed the results within health selection, social support, and artefactual perspectives of inequality.

**Methods:**

Data were extracted from a qualitative project on T1DM lived experiences in southern Ghana. The study participants were young persons living with T1DM (*n* = 28) and their caregivers (*n* = 12). They were purposively recruited to participate in the study using maximum variation and snowball sampling techniques and interviewed in their support group centres, homes, or healthcare facilities using semi-structured interview guides. A computer-assisted qualitative data analysis was performed using QSR NVivo 14 software, and the results were categorised into themes.

**Results:**

Three themes were identified from the transcripts. These themes were *school and classroom attendance*, *choice of school*, and *school/academic performance*. T1DM was a major reason for patients’ limited contact hours with teachers, school drop-out, preference for day schools rather than boarding, opting for vocational training instead of continuation of formal education, limited concentration at school, and delayed educational progression.

**Conclusion:**

T1DM impacted the schooling of young people living with the disease. The mechanisms of these impacts, and young peoples lived experiences are not artefactual, but rather support discourses on health selection and inadequate social support for young people living with the disease. The results call for the need to develop educational and social interventions to address these barriers. The full implementation of the Inclusive Education Policy (IEP) may contribute to reducing educational and social inequalities caused by ill-health.

**Supplementary Information:**

The online version contains supplementary material available at 10.1186/s12889-024-18590-y.

## Introduction

Across the globe, recent estimates show that about 8.4 million individuals are living with Type 1 Diabetes Mellitus (T1DM) [1]. A fifth of these individuals live in low-and-middle-income countries (LMICs) and about 18% of the global average are young people and children (aged below 20 years) [1]. In Ghana, about three thousand young people aged less than 20 years are living with T1DM [2] with a higher proportion that usually go unreported. For this latter group, the condition can be crippling. Associated T1DM health conditions such as Mauriac syndrome, thyroid disorders, and bone ill-health [3, 4] negatively impact growth and development while reducing the probability of survival. Beyond physical growth impairment, the disease can impact cognitive development. Over the past decades, several scholars have provided evidence on the adverse impacts of T1DM on young people’s educational attainment [5–7]; strengthening the need to develop interventions to accommodate such warriors (a lay term used for young people living with T1DM in Ghana) [8] in educational settings. An educational intervention pertinent to students living with T1DM is the Inclusive Education Policy (IEP) which highlights concerns about learner diversities such as “persons with other health impairment and chronic diseases” [9].

There are two major pathways that explain the influence of T1DM on educational attainment among warriors. First, T1DM operates through impaired physical health. Young people living with T1DM require regular medical attention and this leaves them with little time and energy for school and other educational activities [5]. Yu and colleagues found that on average, children with T1DM missed 8.3 more days annually compared to their healthy counterparts [10]. Glaab, Brown and Daneman also attested to more missed days for diabetic children and reported that in Ontario, Toronto, it could be 2 or 3 days more [11]. Second, T1DM is associated with several neuropsychological conditions [7] such as hypoglycaemia, hyperglycaemia, and diabetes ketoacidosis. These conditions affect mental alertness [6, 12] which results in poor overall final grades and delayed educational progression. Given the central role of education in future employment prospects, T1DM can, in the long term, affect their socio-economic positions in life via health selection.

Within the contexts of this study, the cornerstone of health selection is that students’ overall health dictates their social resources needed to excel in school, with those affected by ill health drifting down the educational ladder and those with good health drifting upwards the educational ladder (upward drift) [13–15]. Health selection can be tested using comparative data to examine associations between health and educational outcomes, but it is imperative to contextualise the interpretation of this synergy through subjective experiences. We find this theory important to advance our discussion on T1DM and schooling due to three main reasons. First, T1DM is a life-long condition with no cure and no days off management. Second, it mostly manifest among young people who may be in school and studying towards various socio-economic positions in society. Third, T1DM is a multidimensional condition that affects the cognitive and socio-economic participation of patients.

The Ghanaian education system has three levels of pre-tertiary education. They are the primary, junior high, and senior high school levels. Currently, the government funds public education on all three pre-tertiary levels as part of its “free education” agenda. This helps to reduce the financial burden for people, of which children and student warriors are inclusive. The first two levels operate the day-schooling module where children attend school and return home on a day-to-day basis. Day schools are usually located in communities or towns closer to their place of residence and in easy commute. At the senior high school level, two modules are adopted – day and boarding schooling. The latter requires students to stay in accommodation facilities (mostly referred to as “boarding houses” or “hostels”) within the schools’ premises. A senior high school may adopt either one of the approaches or both. Thus, for these warriors and their caregivers, decisions on the choice of school move beyond pursuing their programmes of study - the accommodation module that can make schooling convenient for them is crucial. At the tertiary level, the situation is further exacerbated by the limited number of public tertiary institutions, high priced private institutions, and the temptation to consider other vocational training options (e.g., apprenticeship) which may not necessarily be in the interest of warriors. There are high trade-offs for warriors in either choosing the best schools/educational institutions or those that are convenient given their health condition – an add-on to inequalities in educational choices and outcomes.

There is limited research on the impact of T1DM on schooling in LMICs including Ghana, and more deplete as discussed within health selection, social support, and artefactual perspectives globally. On one hand, this is partly due to the limited attention T1DM among young warriors has received and on the other hand, limitations in data availability to investigate the impact of T1DM on educational outcomes or perhaps, limited funding to explore such research interests in LMICs. The limitations in this area have implications for both present and future attempts to promote quality education for all, and reduce avoidable educational inequalities. Furthermore, existing studies, though useful, have provided little qualitative evidence to fill in the “how” and “why” and in most instances, the experiences of caregivers are left out. Since caregivers are involved in their children’s educational choices and considerations, their insights and experiences on these issues are indispensable and need to be featured in ongoing discourse.

Several large-scale studies have compared educational and school performance of young people living with T1DM with their non-diabetic counterparts and concluded that T1DM has an adverse impact on the schooling of young people with T1DM [16–18]. In this research, emphasis is placed on the lived experiences of warriors at the primary to tertiary levels of education and their caregivers to understand how T1DM influence educational outcomes. Using qualitative approaches, this research explores the educational experiences of young people living with T1DM to provide more contextualized, African, and diversified evidence on the impact of T1DM on educational outcomes, and highlight evidence which is critical to understanding the linkages between ill-health, education, inequality, and socio-economic development. The evidence from this research will be of great importance to guide educational programs, interventions, and policies that target young people with medical conditions in general, and those with T1DM in particular.

## Materials and methods

### Study area

The study was conducted in southern Ghana, specifically Greater Accra, Central, and Western regions where T1DM is most prevalent [19]. These regions represent a heterogeneous population due to the high migration of people from both the middle and northern belts of Ghana. This zone benefits from improved socio-economic infrastructure including major healthcare infrastructure. For instance, healthcare facilities such as the Korle-Bu Teaching Hospital, Greater Accra Regional Hospital (Ridge Hospital), and Cape Coast Teaching Hospitals are found in this zone and are major referral points for T1DM care [8, 20].

### Study design and participants selection

Data were drawn from a qualitative project on the lived experiences of young persons living with T1DM and their caregivers in southern Ghana. Amongst the participants studied were 28 young persons living with T1DM, and 12 caregivers. The warriors were identified at Diabetes Youth Care (DYC: an NGO that provides monthly psychosocial support to a diverse group of young persons living with T1DM across the regions) support group centres in the study area (n = 13), healthcare facilities (n = 6), and their homes (n = 9). They were purposively recruited to participate in the study using maximum variation, convenience, and snowball sampling techniques. The maximum variation selection techniques relied on the various DYC register, allowing participants with different socio-demographic characteristics such as age, sex, and educational levels to be interviewed. Using snow-ball approaches, participants who were non-members of the DYC/rarely went to the DYC monthly support meetings were followed up in their homes or healthcare centres and interviewed. The inclusion criteria were as follows:

- The warrior must be aged 14–24 years and living in southern Ghana for not less than 24 months.
- The warrior must either be a student at the time of the study or must have been a student within 24 months preceding the study.
- The caregiver should be living with and providing care for the warrior for not less than 24 months. The 24 months were deemed as sufficient time to reduce recall biases. Participants’ selection and data analysis were ongoing, and this helped to gauge the point of saturation/information power.

### Data collection procedure

Qualitative data were collected at the DYC support group centres, participants' homes, and healthcare facilities using a semi-structured interview guide. The interview guide was pre-tested and revised before it was used for data collection which lasted 40 days (1st August 2021 – 9th September 2021). The lead author negotiated access to the network of warriors through the organisation’s leadership across the regions. Three research assistants who have experience in qualitative methods, its philosophies and practices were trained on the issues covered in the interview guide. They visited and interviewed the warriors either during their monthly support group meetings, healthcare facility, or their usual place of abode. The data collection methods were both face-to-face (n = 17) and via telephone (n = 11). The interviews were mostly conducted in the morning and late afternoon, and at an appropriate location agreed upon by participants. A maximum of one interview was conducted in a day. All interviews were conducted in either English, Fanti, Twi, or a combination of these languages as best spoken and understood by participants. The average duration of the interviews was 48 min and were audio-recorded. All study participants received a compensation of a dollar (US$1) after data collection to cover transportation back home, and airtime costs.

The semi-structured interview guide covered four (4) sections (See Supplementary file): Socio-demographic characteristics (Section A), T1DM management knowledge (Section B), effects of T1DM on health and education (Section C), challenges of managing T1DM and coping strategies (Section D). This study extracted data from Section A and C, specifically, the socio-demographic characteristics, and the effects of T1DM on education. In this section, the questions explored included “*What does it mean to be living with T1DM as a student? What have been some of the good and challenging moments you have faced as a student living with T1DM? Has T1DM affected your educational choices in any way, and are there any stories to share?* The caregivers were asked similar questions including *“How has T1DM affected your child’s education".* The interviews were transcribed verbatim into the English language soon after data collection and password protected.

### Rigour

Quality assurance mechanisms were embedded in the planning, fieldwork, and analysis phases. We employed credibility methods such as observation, extensive engagement with participants, data triangulation, and participants re-checking of transcripts. Also, there were multiple field officers and coders, and the use of maximum variation sampling technique was important to collect data from different participants. We adopted a phenomenological study design and a chain of evidence to investigate the same issues. We present participants quote including discrepant information to allow readers to vicariously reflect on the data themselves. The semi-structured interview guides were guided by a systematic review of the literature, and the lead researchers' regular involvement with T1DM patients and carers.

### Data processing, analysis, and presentation

The analytical technique was thematic analysis. The transcripts were coded independently by the field assistants. The Codebook was shared among the researchers, discussed and themes were generated from the codes. A computer-assisted qualitative data analysis was performed using QSR NVivo 14 software. This software facilitated the comparison of different codes via matrix coding. For instance, comparing participants’ age, sex, and place of residence with classroom attendance. Data analysis was ongoing, and codes were inductively identified from the transcripts. Data coders assigned a word or brief statement of description to depict significant statements that related to the study objectives. For instance, all issues such as “*sought permission out of the classroom to check my sugars, take my shots”, I was on admission while class was in session”, I didn’t go to school due to hospital reviews”* were thematised as *limited contact hours with teachers.* In like manner, narratives such as *“I went hypo during examinations, and I couldn’t concentrate during examinations”* were thematised as reduced levels of concentration. These significant statements were categorised into themes. Participants’ information was anonymised using a shared *in-vivo* term they had chosen to call themselves [*warriors, meaning fighting against T1DM*].

### Ethical consideration

Ethical clearance was obtained from the University of Cape Coast Institutional Review Board before the start of the data collection [Clearance ID: UCCIRB/CHLS/2021/19]. To accommodate the health and safety needs of participants amid the global Covid-19 pandemic, the study adhered to the Covid-19 protocols, including a 2-m social distance between interviewers and participants. The leadership of the DYC and caregivers consented for participants below age 18, and the assent of these minors was sought as well. Signed copies of informed consent forms as well as verbal consent were obtained or sought respectively prior to the commencement of data collection.

## Findings

### Background characteristics of participants

Forty (40) participants representing two target groups - patients (warriors) and caregivers were interviewed. Twenty-eight (28) of them were warriors. Within this group, there were equal representation of males and females. Ten of the warriors were aged below 19 years. The average duration of T1DM experience for the warriors was about 8 years. Twelve of them reported an immediate family history of diabetes [undifferentiated], and most (*n*=19) warriors were students. For instance, 11 of the participants had attained Senior High School level education, eight had attained tertiary education, and two had attained primary education. Most of the warriors stayed with their caregivers—mostly their mothers and grandmothers (n = 11), compared with those who stayed with a non-relative (n = 2). All the warriors were actively covered under the national health insurance scheme (NHIS). A warrior was married, and two others were cohabiting. Three of them had newly joined the DYC, and 7 were irregular meeting attendees. The average age of the caregivers was 45 years, mostly with Basic education (JHS: n = 7), Senior High School (SHS: n = 3), and tertiary (n = 1) levels of education. Nine of the 12 caregivers were biological mothers of a warrior, amongst whom some confirmed they have DM including T1DM. Eight of the caregivers were married and mostly engaged in petty trading. The caregivers have been engaged in T1DM care for about 6 years. Table 1 summarises the socio-demographic characteristics of the study participants.

**Table 1:** Socio-demographic characteristics of participants

Participant Characteristics	Participant Category		Total (*N* = 40)
	**Warriors (*n* = 28)**	**Caregivers (*n* = 12)**	
**Sex**			
Female	14	11	25
Male	14	1	15
**Age group (in years)**			
14 – 19	10	-	10
20 – 24	18	-	18
30 – 39	-	4	4
40 – 49	-	3	3
50 and above	-	5	5
**Duration of living with/providing T1D care**			
< 5 years	5	4	9
5–10 years	17	6	23
> 10 years	6	2	8
**Highest educational level**			
Never Attended	-	1	1
Primary	2	4	6
JHS	7	3	10
SSS/SHS	11	3	14
Tertiary	8	1	9
**Family history of DM**			
Yes	12	7	19
No	13	5	18
Don’t know	3	-	3
**Primary caregiver (PCG)**			
None	6	-	6
Both Parents	3	-	3
Mother/Grandmother	11	-	11
Father	3	-	3
Other relatives	3	-	3
Non-relative	2	-	2
**PCG Occupation**			
Salary earner	-	2	2
Petty trader	-	8	8
Unemployed	-	2	2
**Religious affiliation**			
Christian	25	11	36
Muslim	3	1	4

### Themes on the effect of type 1 diabetes (T1DM) on patients’ schooling

Three themes were identified from the inductive analysis. These themes were school and classroom attendance, choice of school, and school/academic performance. The first theme [school and classroom attendance] was formed from sub-themes on limited contact hours with teachers and school drop-out. The second theme [choice of school] was formed from sub-themes on day versus boarding, and opting for vocational training. The final theme [school/academic performance] was formed from sub-themes on limited concentration and delayed educational progression.

### School and classroom attendance

The first theme concerned school and classroom attendance of young persons living with T1DM. Type 1 diabetes affected young person’s school and classroom attendance. Concerning this theme, two main sub-themes were identified. These sub-themes were limited contact hours with schoolteachers and early school drop-out.

### Limited contact hours with schoolteachers

Young people living with T1DM faced limited contact hours with teachers during school sessions. This sub-theme was formed from two main issues which were *frequent permission out of the classroom, and absenteeism*. Young persons living with T1DM sought permission outside the classroom to self-monitor their blood glucose, inject their insulin or urinate frequently while classes were in session. In explaining this issue, this was what was said:

*“I will usually go out during class sections to urinate or seek permission to go and check my sugar or something”* [a 20-year-old female warrior with 6 years of lived experience].

A 21-year-old male warrior with 10 years of lived experience corroborated this issue by sharing his experience while writing his final examination in high school. He revealed how T1DM disturbed his concentration while writing his examinations:

*“My condition disturbed me a lot when I was writing my WASSCE [West African Senior School Certificate Examination]. I couldn’t concentrate especially when my sugars were going low, and I had to be getting out to urinate and check my sugars amidst the limited time pressure”.*

Concerning *absenteeism*, most young persons living with T1DM revealed that they usually absented themselves from school or classroom because of T1DM-related hospital/school-based infirmary admissions, caregiver delays in preparing food for them early enough to go to school, sick days, and T1DM complications. Excerpts from participants support this finding:

*“I had to miss days of school to get check-ups and medicines from the hospital which made my education a bit off the rail because by the time I would return from the hospital, school will almost be over”* [a 21-year-old male warrior with 7 years of lived experience].

Assertions from some caregivers corroborate this finding. For instance, in the words of a 50-year-old mother with child who had 7 years of lived experience, this was what was said:

*“Since 2014, there are several times that he was admitted to the hospital and therefore couldn’t write examination or didn’t have the time to study for it”.*

### Early school dropout

The second sub-theme on school and classroom attendance was early school drop-out due to T1DM and its complications. Some warriors dropped out of school due to T1DM complications and its related stigma. Specifically, eye and foot complications were common reasons for some patients dropping out of school. This was common amongst patients from poor socio-economic backgrounds. In explaining the issue of complications, this was what was said:

*“I stopped school at class 6 because of my condition. I found it difficult to read from the board. The school’s food wasn’t good for my condition, and this wound [has an impaired wound healing] so it was stressful at school”* [a 15-year-old female warrior with 5 years of lived experience].

Also, some young persons dropped out of school due to T1DM-related stigma. They experienced stigma concerning emaciation, insulin injection, and name-calling as always “*sick*”. Excerpts from participants support this finding.

*“My friends tease me [starts to cry], they tease me and tell me that I am a sickler”. My … [same religion] friends who know that I inject myself don’t laugh at me, the other [religion] laugh at me. I couldn’t stay in the school, and my mother didn’t have money to change my school, so I stopped”* [a 17-year-old female warrior with 5 years of lived experience]*.*

### Choice of school

The second theme concerned the choice of school. This was found to be a major issue of concern for young persons living with T1DM and their caregivers. Issues pertinent to the choice of school were day versus boarding and opting for vocational training.

### Day versus boarding

From the outset, caregivers selected secondary schools for their children living with T1DM to attend. These schools were either close-by their place of residence, or caregivers had personal contact with the school authorities intended for their children to attend. In explaining these issues of proximity and personal contacts at the school, this was what was said by a 55-year-old mother with 5 years of lived experience:

*“She had admission at* [mentions name of school] *but due to her condition and distance, I obtained* [mentions name of school] *SHS for her because it is closer, and her sister went there and knows a woman who stays close to the school, so I took my child into her house to be under her care”.*

Caregivers were hesitant about allowing their children living with T1DM to be in boarding schools due to concerns over limited T1DM support in school. Excerpts from caregivers and young people living with T1DM showed that the boarding house compromised their adequate management of T1DM. As such, these students living with T1DM commuted to and from school, rather than being in the boarding house unlike most of their colleagues without diabetes. In their narrations, this was what was said:

*“Those I completed JHS with are in the boarding house. I couldn’t go to the boarding house due to my condition…I would have loved to go the boarding house also because of improved study time, but that would be difficult for me”*. [a 17-year-old female warrior with 5 years of lived experience].

*“I have not chosen the school [SHS] yet but my mother says I will go to a day school so that she can have time for me”* [a 17-year-old male warrior with 4 years of lived experience].

In explaining this issue*,* a 41-year-old mother with 5 years of lived experience provided insights to corroborate this finding by revealing this:

“*One of my major concerns is that I can’t allow her to go to boarding school. She may sleep and something may happen to her, and no one will know”.*

For some students whose caregivers were concerned about their inability to properly manage their condition while transitioning from SHS to the University, they preferred that their children spend the first year of their university education at the house to gather enough time to experience the university environment prior to transitioning fully to campus residence. In their revelation, a 21-year-old female warrior with 7 years of lived experience had this to say:

*“In level 100, I was moving from home to school. They [parents] wanted to be sure that I will be able to cope and manage my condition in school before allowing me to move a year after. My daddy used to drop me off every day at school before going to work”.*

### Opting for vocational institutions

Due to the worries about the boarding house, a few young people living with T1DM shunned the Senior High School system, with its boarding provisions to enrol in vocational institutions. Concerning this, a 20-year-old female warrior with 5 years of lived experience had this to say:

*“My uncle was like going to the boarding house was a whole lot so I enroled in a fashion school (vocational institute) where I am doing clothing for two years… I always wanted to be a journalist, but I think circumstances, so I was like okay, doing clothing is also a job”.*

In buttressing this issue, a 53-year-old caregiver with 3 years of lived experience shared her plans about enrolling her warrior into a vocational institution. In her revelations, this was what was said:

*I am taking care of her to finish her BECE [Basic Education Certificate Examination] and I plan of asking her to learn a trade or start some vocational school.*

### School/academic performance

There were concerns about the effect of T1DM on patients’ school performance (defined as meeting goals and objectives set for school). We found.

that, due to the effects of T1DM on school attendance, caregivers attributed the poor academic performance of their warriors to the demanding nature of T1DM management. They shared that T1DM affected their wards’ academic performance through polyuria and limited concentration during examinations. The sub-themes identified were *limited concentration* and *delayed educational progression*.

### Limited concentration

Participants shared their experiences with living with T1DM while writing examinations, particularly the WASSCE (West African Senior School Certificate Examination). In their revelations, T1DM was the major reason for their lack of concentration due to severe low blood glucose and polyuria. In elucidating this issue, this was what was said:

*Diabetes affected me in many ways, I remember I was so worried about my WASSCE. I remember I went hypo [hypoglycaemia] on the very first day we started WASSCE* [a 24-year-old male warrior with 13 years of lived experience].

In buttressing this issue, a 48-year-old mother with 11 years of lived experience said this:

*“When she sat for WASSCE, she didn’t do well because she was sick from her condition and said she couldn’t concentrate due to very low sugar levels”*.

### Delayed educational progression

There were concerns held by participants about their delayed educational progression. This concern was also shared by the parents/guardians of the young persons living with T1DM. Concerning educational progression, a 48-year-old mother with 11 years of lived experience had this to say:

*“All her friends will be completing University this year, she’s the only one who just enroled”.*

In buttressing this issue, a 56-year-old mother with 6 years of lived experience with two children living with T1DM said this:

*“She is 19 years, but her brain is dull. It is now that she’s picking up some behaviours from time to time. Her junior brother is in SHS, and she is in JHS”.*

## Discussion

We studied the influence of T1DM on schooling by exploring the experiences of young people living with T1DM and their caregivers in Ghana. The results show that T1DM impacted the schooling of young persons living with the disease in adverse ways. T1DM impacted young person’s schooling through absenteeism/limited classroom attendance, choice of school, and school/academic performance. These findings add new knowledge regarding how diabetes impacts on education among young people living with the disease – a critical pathway to socio-economic development. The results further provide a considerable amount of evidence to explain the various pathways to a downward drift in educational settings.

Concerning school and classroom attendance, the finding that T1DM contributes to students’ absenteeism due to increased hospitalisation or medical appointments has been confirmed in Australia [21] and Canada [11]. By its nature, the management of T1DM is complex and demanding [22]. It requires patients and their caregivers to make daily lifestyle modifications which can be overwhelming. Also, poor management of T1DM can lead to acute complications such as hypoglycaemia and diabetes coma which requires immediate hospitalisation [23, 24]. As confirmed in a related study, the experience of T1DM-related stigma is a major reason for missing school days [22]. Due to polyuria, use of glucose monitoring devices and insulin injection outside of classroom, young people living with T1DM frequently miss class sessions [18, 25]. These evidence demonstrates the impact of T1DM on school and classroom attendance and calls for the need to develop interventions to address these barriers of schooling.

The finding that T1DM influenced the choice of school for warriors has been reported in other studies. The fear of allowing children living with T1DM to be in boarding schools or stay close to caregivers was emphasised. This is consistent with the report by Kratzer that parents fear that their children’s diabetes will not be managed properly while away from home [26]. Caregivers, therefore, want to keep close relations with their children [27, 28]. For warriors in Senior High and Tertiary levels of schooling, the prospects of transitioning to boarding school/higher education was a daunting panorama for many of the caregivers as it meant moving from a safer space [home] to an unsafe environment [school] for T1DM management. Although, some warriors felt the need to be in the boarding house largely due to reasons including *improved study time*, T1DM and the complexities of management constrained both patients and their caregivers to normalise avoiding the boarding school system, and opting for being a day student or apprenticeship. Shunning the boarding house was primarily a caregiver’s decision, informed by the constraints of group quarter housing - poor diet, supervision, and healthcare accessibility, and therefore geared towards providing safer spaces for the adequate management of T1DM.

**Fig. 1:**
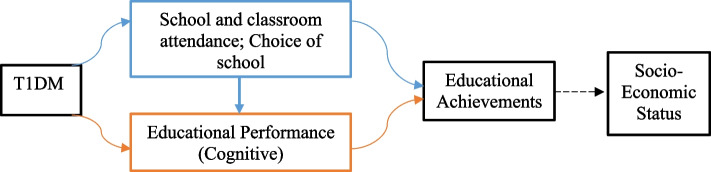
Pathways through which T1DM may affect educational achievements and plausibly SES.  Source: Owusu B.A and Doku D.T., (2024)

Some previous quantitative studies illustrate that T1DM has implications on education and academic performance [29, 30]. As found in this study, TIDM affected not only daily activities but also impinged on their academic performance, concentration on schoolwork, and school progression [31]. Ahmed et al. found similar results in Khartoum (Sudan) on the implications of T1DM on academic progression of diabetic school children [32]. In their meta-analysis, Brands et al., found that warriors have a significantly lower cognitive performance, and obtained significantly lower academic grades compared with their non-diabetic classmates [16, 33]. In Sweden, Persson and colleagues reported that the mean final grades from compulsory school were lower for child warriors and only a handful were able to progress to upper secondary education [6]. In a related study, T1DM was reported to affect warriors’ choice of academic programmes - warriors choose academic programmes that may offer them insights into their T1DM including nutrition programmes[8]. Decisions on school, academic progression or career choices provides individuals with the requisite knowledge and skillset for the next level of employment [34]. Choice of school and career as evident in this study raises concerns about the career pathway, socio-economic participation, and satisfaction of warriors about their career prospects. Given that career choices explicitly train people for various employment prospects, there can be high trade-offs. Evidence from Scotland also revealed additional characteristics that underlie the low grades among young warriors. They include absenteeism, learning difficulties, and to a greater extent, school exclusion [5]. Thus, a drift hypothesis can occur among young people such that T1DM can compound health, academic, career, employment, and income constraints which can influence their socio-economic prospects and positions in life (Fig. 1).

Insofar as the health selection is true, this would imply that the social consequences of ill-health will need to be greater among young persons living with T1DM who suffer the brunt of their condition, and that they are more likely to be found amongst the lower socio-economic positions or groups. Despite the lack of a comparison group (young people without T1DM), the experiences of young persons living with T1DM with respect to the impact of T1DM on their schooling or education is not artefactual. Certainly, the analysis of different datasets and application of different measures produces different results, but further supports underlying concepts being tested. These theoretical insights (health selection or artefact) are rather speculative and therefore limited; none of them wholly provide a holistic explanation of inequality. For adolescents living with chronic T1DM, particularly those in senior and junior high schools, this “sick role” can be an “opportunity” to miss school days. Also, since students in boarding houses relatively enjoy stable study times, group studies, and adequate academic supervision, missing boarding schools can blur the academic achievement of warriors, and their progression to higher levels of education in the Ghanaian context. However, in some instances, educational inequalities such as those found in this study are structural and therefore preventable.

Education in Ghana is a basic human right for all citizens. The overarching goal of the Inclusive Education Policy (IEP) is to “*redefine and recast the delivery and management of educational services to respond to the diverse needs of all learners* with special educational needs *…”* including *“…those children who are failing in school because they experience barriers that prevent them from achieving optimal progress in their learning and development*” ([9], p3-4). T1DM is a barrier to adequate learning, progress, and development of warriors as it adversely affects their school attendance, performance, choice of school and cognitive abilities. The results of this study underscore the need to, as part of a wider educational reform, be aware of the different health needs of learners, especially, those suffering from disruptive health conditions such as T1DM, its implication on learners’ educational achievements, and equitably address them. This will entail all stakeholders, including teachers, school authorities, school nurses, kitchen staff, working together to respond to the peculiar needs of warriors that may arise/be pertinent in the school setting.

## Strengths and limitations of the study

To the best of our knowledge, this is the first study in Ghana that focuses on the impact of T1DM on schooling by exploring the experiences of warriors and their caregivers. This study is therefore an add-on to T1DM studies which are limited in Ghana and LMICs. The results also highlight the voices of warriors and their caregivers, and make arguments towards a potential health selection - requiring a targeted, age-appropriate and systemic interventions to address these mechanisms of impact. These aside, there are some methodological limitations worth mentioning. First, there was no comparison group (either within group or young people without diabetes) to enhance deeper understanding of the observed differences in school outcomes. Also, the study could have benefited from comparing the school attendance and academic results of warriors to their classmates without T1DM. Thus, the discussion on inequality is rather speculative, and will require further quantitative studies, also to test Fig. 1 as a plausible pathway. Consequently, the conclusion of this study can be generalised only to sub-groups within our inclusion criteria.

## Conclusion

T1DM was a major reason for warriors absenteeism, limited contact time with schoolteachers, choice of school, school drop-out, and limited concentration at school. These findings are not artefactual, but seem in support of health selection and inadequate social support perspectives. Addressing these barriers of schooling for young people with T1DM will require the implementation of the inclusive education policy in Ghana. Such targeted intervention in the life of young people would most likely break the continuous linkage between ill-health and social class. Also, comprehensive diabetes education programmes for young persons living with T1DM are essential to build upon their coping strategies, improve glycaemic control as it affects school outcomes, and improve caregivers' trust and confidence in teachers and school management’s capability to support their children with T1DM while in school.

### Supplementary Information

**Supplementary Material 1.**

## Data Availability

The transcribed data and/or analysed during the current study is available upon request from the Department of Population and Health, UCC at pop.health@ucc.edu.gh.
