# Supplementary material for: Living with type 1 diabetes and schooling among young people in Ghana: a truism of health selection, inadequate support, or artefactual explanation of educational inequalities?
Source: BMC Public Health. 2024 Apr 24;24:1137. doi: 10.1186/s12889-024-18590-y (PMC11040772; doi:10.1186/s12889-024-18590-y)
Supplement: Supplementary file 1 — Supplementary Material 1. [file 12889_2024_18590_MOESM1_ESM.docx]

**LIVED EXPERIENCES OF YOUNG PERSONS WITH TYPE 1 DIABETES AND THEIR CAREGIVERS IN SOUTHERN GHANA**

**IN-DEPTH INTERVIEW GUIDE FOR YOUNG PERSON’S**

| **SECTION A: Socio-demographic characteristics of participants**  Could you please tell me about yourself? Ask about | |
| --- | --- |
| 1. *Age* | 1. *Sex* |
| 1. *Duration of diabetes* | 1. *Religious affiliation* |
| 1. *Place of residence* | 1. *Marital status* |
| 1. *Level of education* | 1. *Occupation* |
| 1. *Primary caregiver* | 1. *Family history of diabetes?* |
| 1. *Caregiver’s Occupation* | 1. *Insulin injection per day* |
| 1. *Telephone number* | 1. *Interview start date* |
| 1. *Interview end date* | 1. *Duration of interviews* |
| 1. *Interviewer* | 1. *Etc.* |

| **SECTION C: Effects of diabetes on young people’s health and well-being** |
| --- |
| **PREAMBLE:** Diabetes affects several areas of individuals’ health and socioeconomic lives. This section seeks to solicit for information about how diabetes has affected your health including any known complications as well as other facets of your socio-economic life. |

1. Which areas of your life has diabetes affected and why? [if not mentioned,
   ask about education/work, friendship, relationship etc.]
2. Kindly tell me about how diabetes has affected your social activities? [Probe:
   effect on activities of daily living, sleep, school/work, friendship/relationship, family conflicts, choice of games etc.]
3. How have your school/workplace related with you since your diagnosis?
4. What does it mean to be living with T1DM as a student?
5. What have been some of the good and challenging moments you have faced as a student living with T1DM?
6. How has T1DM affected decision making towards your education? Are there any stories to share?

**Closing courtesies**

Thank you for your time and information. Can I please come back or call you for additional information if there is the need?

**NB: Sections which were not directly related to this paper are omitted.**

**INTERVIEW GUIDE FOR PARENTS/CAREGIVERS**

1. Can you tell me about yourself: *[Age, sex, occupation, place of residence,
   family history of diabetes, income, educational level, marital status, parity,
   years involved in T1D care?]*

| **SECTION C: Effects of diabetes on young people’s health and well-being** |
| --- |
| **PREAMBLE:** Diabetes affects several areas of individuals’ health and socioeconomic lives. This section seeks to solicit for information about how diabetes has affected your child’s health and education. |

1. How has diabetes affected your child/children? [*Ask for any known complications, impact on education etc.]*a. Ask participant to share a story if any.
2. How has T1DM affected your child’s education?
3. How are decisions made concerning your child’s education/schooling?

**Closing courtesies**

Thank you for your time and information. Can I please come back or call you for additional information if there is the need?

**NB: Sections which were not directly related to this paper are omitted.**
